# Role of procalcitonin and high-sensitivity C-reactive protein in sepsis: a prospective study

**DOI:** 10.1186/cc10394

**Published:** 2011-10-27

**Authors:** S Das, D Anand, S Ray, S Bhargava, A Manocha, M Kankra, LM Srivastava

**Affiliations:** 1 Department of Biochemistry, Sir Ganga Ram Hospital, New Delhi, India; 2 Department of Critical Care Medicine, Sir Ganga Ram Hospital, New Delhi, India

## Introduction

Sepsis is the most common cause of morbidity and mortality in ICU patients. The clinical signs of infection and routine laboratory tests are not specific and at times misleading. Even the bacteriological evidence of infection is not sensitive enough. In view of this diagnostic and therapeutic dilemma, an effective and specific marker is needed that can support or exclude the diagnosis of infection. Hence we evaluated the usefulness of biochemical markers such as procalcitonin (PCT) and high-sensitivity C reactive protein (hsCRP) in monitoring the therapeutic response to treatment in patients with sepsis.

## Methods

Fifty-seven patients admitted to the ICU of Sir Ganga Ram Hospital, New Delhi, India from July 2010 to April 2011 with a fresh episode of sepsis were included in the study. PCT and hsCRP were analyzed in serum samples at 0 hours, 24 hours and 72 hours. Blood cultures were performed on the day of admission (0 hours) and the patients were categorized as culture positive or culture negative. Patients were followed up for 28 days and were then grouped as survivors and nonsurvivors.

## Results

During the observation period of 28 days, 44 patients survived and 13 expired. Over a period of 0 to 72 hours the PCT level decreased in 86% survivors as compared with 46% nonsurvivors (*P *= 0.001), whereas it increased in 13.6% survivors as compared with 53.8% nonsurvivors (*P *= 0.001). The change in the levels of hsCRP in both surviving and nonsurviving patients was not significant. The 0-hour blood culture was positive in 24 out of 57 patients. The PCT levels at 0 hours was significantly high in culture-positive patients (*P *= 0.002) as compared with culture-negative patients, whereas there was no significant difference in the hsCRP levels in the above-mentioned groups. The area under the curve of PCT and hsCRP in culture-positive and culture-negative patients was 0.743 (95% CI = 0.608 to 0.878, *P *= 0.002) and 0.564 (95% CI = 0.414 to 0.715, *P *= 0.405), respectively.

## Conclusion

These observations indicate that PCT is a better prognostic indicator than hsCRP and is also a better predictor of bacteremia in the newly admitted critically ill patients of sepsis. Hence, routine use of PCT as a monitoring tool may aid in appropriate therapeutic intervention in patients with sepsis.

